# Heel-Rise Test for functional assessment in chronic venous insufficiency: a validity study

**DOI:** 10.1590/1677-5449.202501772

**Published:** 2026-09-07

**Authors:** Marina Silva Reis, Keity Lamary Souza Silva, Antonielly Rocha de Souza Pereira, Vivian Camargo Chaves, Stefânia Guimarães Nery, Dalyla Lemos de Souza Silva, Pedro Henrique Scheidt Figueiredo, Henrique Silveira Costa

**Affiliations:** 1 Universidade Federal dos Vales do Jequitinhonha e Mucuri – UFVJM, Diamantina, MG, Brasil.

**Keywords:** venous insufficiency, muscle strength, muscle weakness, muscle fatigue, insuficiência venosa, força muscular, fraqueza muscular, fadiga muscular

## Abstract

**Background:**

Chronic venous insufficiency (CVI) affects the superficial and/or deep venous system and is often associated with valve abnormalities and calf pump dysfunction, including reduced lower limb strength. Assessment of calf muscle strength should be part of the clinical routine for these patients. The Heel-Rise Test (HRT) has been proposed for this purpose, but its correlation with other functional instruments remains unclear.

**Objectives:**

To verify the validity of the HRT for assessment of calf muscle strength and endurance in patients with CVI by comparing it with other functional tests.

**Methods:**

Fifty-two patients with CVI (mean age of 67.2±13.2 years, CEAP 1 to 6) were evaluated. All patients undertook the HRT and were instructed to perform maximal plantar flexions until exhaustion. The number of repetitions and the time taken to complete the test were recorded. All Sit-to-Stand test variants (STS5, STS10, STS30, STS60) were also administered. Handgrip dynamometry, the Human Activity Profile (HAP), and the CIVIQ-14 questionnaire were also administered. Correlations were analyzed using Pearson or Spearman tests.

**Results:**

HRT repetitions correlated significantly with STS5 (r = -0.676), STS10 (r = -0.701), STS30 (r = 0.691), STS60 (r = 0.697), handgrip dynamometry (r = 0.275), HAP (r = 0.457), and CIVIQ-14 (r = -0.646). However, HRT execution time was not correlated with handgrip dynamometry or CIVIQ-14.

**Conclusions:**

The HRT is related to calf muscle strength, endurance, and functionality in patients with CVI and should be used for functional assessment of these patients.

## Resumo

**Contexto:**

A insuficiência venosa crônica (IVC) acomete o sistema venoso superficial e/ou profundo e está frequentemente associada a alterações valvares e à disfunção da bomba muscular da panturrilha, incluindo redução da força dos membros inferiores. Avaliação da força da musculatura da panturrilha deve fazer parte da rotina clínica desses pacientes. O teste de elevação do calcanhar (HRT) foi proposto para essa finalidade, mas sua associação com outros instrumentos permanece incerta.

**Objetivos:**

Verificar a validade do HRT na avaliação da força e resistência da musculatura da panturrilha em pacientes com IVC, comparado esse com outros testes funcionais.

**Métodos:**

Foram avaliados 52 pacientes com IVC (idade média de 67,2±13,2 anos, classificação CEAP de 1 a 6). Todos realizaram o HRT e foram orientados a executar flexões plantares até a exaustão. O número de repetições e o tempo de execução foram registrados. Também foram aplicadas variações do teste de sentar e levantar (STS5, STS10, STS30, STS60), dinamometria de preensão palmar, Perfil de Atividade Humana (PAH) e questionário *Chronic Venous Insufficiency Quality of Life Questionnaire-14* (CIVIQ-14). As correlações foram analisadas pelos testes de Pearson ou Spearman.

**Resultados:**

O número de repetições no HRT apresentou correlação significativa com os testes STS5 (r = -0,676), STS10 (r = -0,701), STS30 (r = 0,691), STS60 (r = 0,697), dinamometria de preensão palmar (r = 0,275), PAH (r = 0,457) e CIVIQ-14 (r = -0,646). No entanto, o tempo de execução do HRT não apresentou correlação com dinamometria de preensão palmar, nem com CIVIQ-14.

**Conclusões:**

O HRT está relacionado à força, à resistência e à funcionalidade da musculatura da panturrilha em pacientes com IVC, devendo ser utilizado para avaliação funcional desses pacientes.

## INTRODUCTION

Chronic venous insufficiency (CVI) is a progressive condition characterized by dysfunction of the venous system of the lower limbs, impairing venous return to the heart. It results from structural or functional changes in the veins, such as valvular insufficiency, venous obstructions, or both, causing sustained venous hypertension.^1^

The Clinical-Etiology-Anatomy-Pathophysiology (CEAP) classification is the most widely used system for evaluating chronic venous insufficiency (CVI). The system categorizes patients into classes 0 to 6. In its early stages, patients often report leg heaviness, pain, burning sensations, and fatigue, which typically worsen by the end of the day or after prolonged standing.^2^ As the disease progresses, visible signs such as edema, telangiectasias, varicose veins, and skin changes including hyperpigmentation, lipodermatosclerosis, and ochre dermatitis become more apparent. In severe cases, CVI may lead to venous ulcers that are difficult to heal and prone to recurrence.^3^

The disease affects millions of people worldwide.^4^ In a systematic review with pooled prevalence analysis,^5^ the overall prevalence rates for each clinical manifestation were as follows: C0: 9%, C1: 26%, C2: 19%, C3: 8%, C4: 5%, C5: 1%, and C6: 0.42%. The prevalence of CVI manifesting with varicose veins was also reported by geographic region, with North America having the highest prevalence (23%), followed by South America (22%), while Africa had the lowest prevalence (5.5%). In addition to clinical changes, some authors suggest that patients with CVI also present significant functional impairments.

Functional impairments include reductions in balance and gait speed,^6,7^ muscle strength,^8^ and ankle range of motion (ROM),^9^ which collectively compromise daily activities, work capacity, and quality of life. Calf muscle strength, in particular, is frequently reduced due to both inactivity related to discomfort and structural changes caused by chronic venous hypertension.^10^ Additionally, these patients’ ability to perform daily tasks is limited by reduced muscle endurance.^11^

Given the key role calf muscles play in venous return, it is essential to assess their strength and endurance. The Heel-Rise Test (HRT) is a practical, low-cost tool used for this purpose.^12^ The test involves repeated bilateral heel raises, during which the number of repetitions and the time to fatigue are measured.

In patients with CVI, HRT results, age, and physical activity level have been shown to explain 47% of the variation in CEAP classification (p < 0.001).^13^ One study^14^ demonstrated the HRT’s predictive capacity for functional performance (r^2^ = 0.60), particularly valuable in patients classified as CEAP C3 to C6.

Despite its potential, few studies have investigated the relationship between HRT performance and functional parameters in CVI. Given its simplicity and clinical relevance, the HRT is a promising tool for assessing lower limb function and guiding evidence-based interventions. Therefore, the present study aimed to examine the association between HRT performance and muscle strength parameters in individuals with CVI. We focused on the functional dimension of calf muscle performance in patients with clinically diagnosed CVI, rather than on differentiating specific anatomical patterns of venous disease.

## METHODS

### Study design

This cross-sectional study used prospectively collected data, with participants evaluated at a single time point according to a predefined protocol. The study was approved by the Institutional Research Ethics Committee (CAAE: 31695520.5.0000.5108, approval No. 4.048.767) and all participants provided written informed consent. We followed the STROBE (Strengthening the Reporting of Observational Studies in Epidemiology) statement for reporting.^15^

Participants were individuals of both sexes, aged 50 to 80 years, with a prior medical diagnosis of CVI. Exclusion criteria included any injury or medical condition that could interfere with test performance, such as heel spurs, plantar fasciitis, knee osteoarthritis, or a history of stroke.

Sample size was calculated using G*Power software (version 3.1), based on data from a pilot study involving 10 participants. Assuming a two-tailed test, a correlation coefficient of 0.55 between HRT and the 60-second sit-to-stand test (STS60), an alpha of 0.05, and 95% statistical power, the required sample size was estimated as 52 participants.

### Procedures

Assessments were conducted on different days between September 2024 and April 2025. A detailed anamnesis was performed to identify the clinical and demographic characteristics of the volunteers. Participants were classified according to the CEAP classification.^16^ The CEAP classification categorizes CVI from C0 to C6, ranging from no visible signs (C0), telangiectasias/reticular veins (C1), varicose veins (C2), edema (C3), skin changes (C4), healed ulcer (C5), to active venous ulcer (C6). Cases of chronic venous insufficiency (CVI) were classified as mild (CEAP 0 to 3) or severe (CEAP 4 to 6). After the initial contact, patients performed the HRT, Sit-to-Stand test, and handgrip dynamometry and answered the Human Activity Profile (HAP) and 14-item Chronic Venous Insufficiency Questionnaire (CIVIQ-14).

### Heel-Rise Test

The HRT was performed according to the protocol established by Monteiro et al.^12^ Participants were tested barefoot, standing upright, and bearing weight on both feet. The participant’s dominant hand was placed against the wall in front of them for balance support, with the elbow slightly flexed. A custom-made device was used to ensure maximum range of motion during plantar flexion.

This device consisted of a rod fixed to the wall, designed to determine the participant’s maximum range of motion prior to testing. During plantar flexion, participants were instructed to make head contact with the rod, thereby confirming that maximum amplitude had been attained.

To standardize execution, participants first performed a full plantar flexion, with the metatarsophalangeal joints touching the support surface. At the peak of this movement, the examiner marked the maximum head height on a vertical rod fixed to the wall. The device used consisted of a vertical rod rigidly fixed to the wall in front of the participant, extending above head level, with a movable marker (Figure 1). Participants were instructed to touch their head to this marker on each repetition to ensure full range of motion. The evaluator demonstrated the procedure beforehand and provided clear instructions. During the test, participants performed as many heel raises as possible, to the point of voluntary fatigue, in the shortest possible time. The examiner recorded both the number of repetitions and the total time taken for execution. No verbal encouragement was given beyond the initial instructions. The test was interrupted if the participant failed to keep the knees extended or failed to touch the head to the marker in two consecutive repetitions. The primary outcomes were the total number of repetitions and test duration.

**Figure 1:**
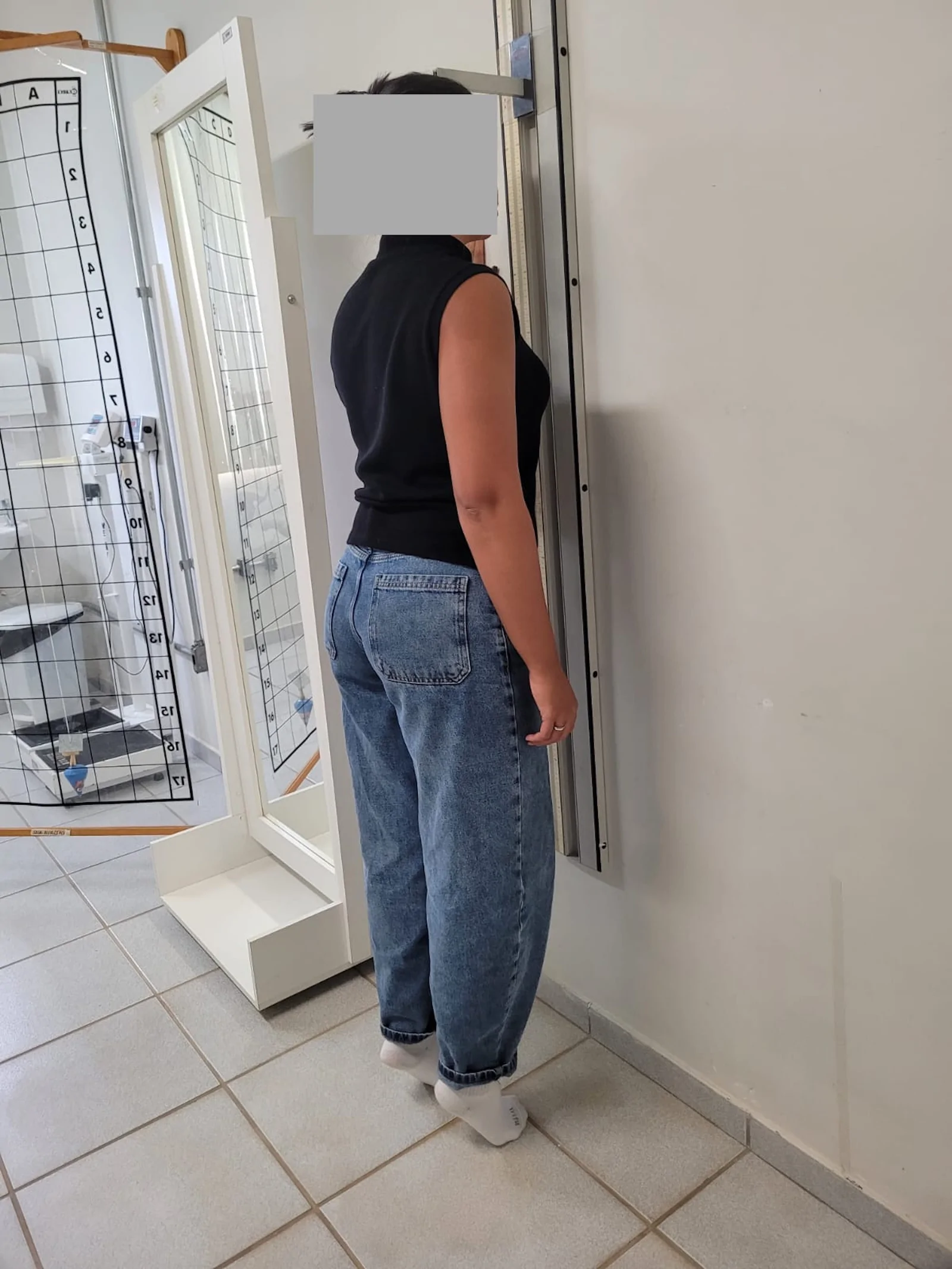
Positioning of the participant and custom-made device used to standardize maximal plantar-flexion amplitude during the bilateral Heel-Rise Test. The rod is fixed to the wall, and the horizontal bar is adjusted to the participant’s forehead height at maximal plantar flexion. During the test, participants are instructed to lightly touch the bar with their forehead at the top of each repetition while keeping one hand on the wall for balance.

### Sit-to-Stand Test

The STS measures lower limb muscle strength and/or endurance. To perform this test, the patient was instructed to sit down and stand up from a standard height chair (46-48 cm) without armrests, positioned against a wall. During the test, the knees and hips were flexed 90 degrees, with the feet flat on the floor and hip-width apart. The hands remained on the hips, without any support.^17^ Four variants were performed: 5 repetitions (STS5), 10 repetitions (STS10), 30 seconds (STS30), and 1 minute (STS60). In the 5- and 10-repetition variants, the time taken for the patient to complete 5 and 10 repetitions, respectively, of sitting down and standing up from the chair was recorded. In the 30- and 60-second variants, the number of repetitions performed during each interval was recorded.

### Handgrip dynamometry

Handgrip strength was determined using a JAMAR® hydraulic dynamometer, following recommended protocols.^18^ Briefly, the patient performed the test seated, with the elbow at 90 degrees of flexion, using the dominant hand, and was instructed to hold the dynamometer as firmly as possible. Standardized verbal commands were used throughout the procedure. The variable of interest was the strength measured in kilograms (kg), taking the largest of the three measurements for the dominant limb.

### Human Activity Profile

The HAP is a 94-item questionnaire that assesses the patient’s level of physical activity based on energy expenditure during daily activities in CVI.^19^ The questionnaire was read to the patient, who answered for each activity described “never did”, “stopped doing”, or “still do”. The variable of interest was the Maximum Activity Score.^20^ The Maximum Activity Score is calculated from the highest-ranked activity that the participant reports still performing.

### Chronic Venous Insufficiency Questionnaire

Health-related quality of life was measured using the CIVIQ-14. According to a previous systematic review,^21^ this questionnaire demonstrated the most robust psychometric properties among health-related quality of life questionnaires for patients with CVI. The CIVIQ-14 comprises 14 questions, distributed across three dimensions: pain (3 items), physical aspects (5 items), and psychological aspects (6 items), which address factors such as daily activities, sleep quality, pain, and emotional distress. The total score ranges from 0 to 70, with higher scores indicating worse quality of life.^22^

### Statistical analysis

Statistical analysis was performed using the Statistical Package for the Social Sciences (SPSS), version 25.0. Data normality was verified using the Kolmogorov-Smirnov test. Continuous variables were presented as mean and standard deviation or median and interquartile range. Categorical variables were presented as absolute numbers and percentages.

Correlation analysis was performed using the Pearson or Spearman tests. Coefficients were classified as correlation absent (r between 0 and 0.10), weak (r between 0.11 and 0.39), moderate (r between 0.40 and 0.69), strong (r between 0.70 and 0.89), or very strong (r between 0.90 and 1.0).^23^

## RESULTS

Fifty-two patients with CVI were assessed and no participants were excluded from the study. Figure 2 shows a flowchart illustrating patient selection. Most patients were female, and 26.9% were classified as CEAP class 4 or higher. The mean number of repetitions and time taken for the HRT were 41.3±21.1 repetitions and 42.4±17.2 seconds. Table 1 lists the sample characteristics.

**Figure 2:**
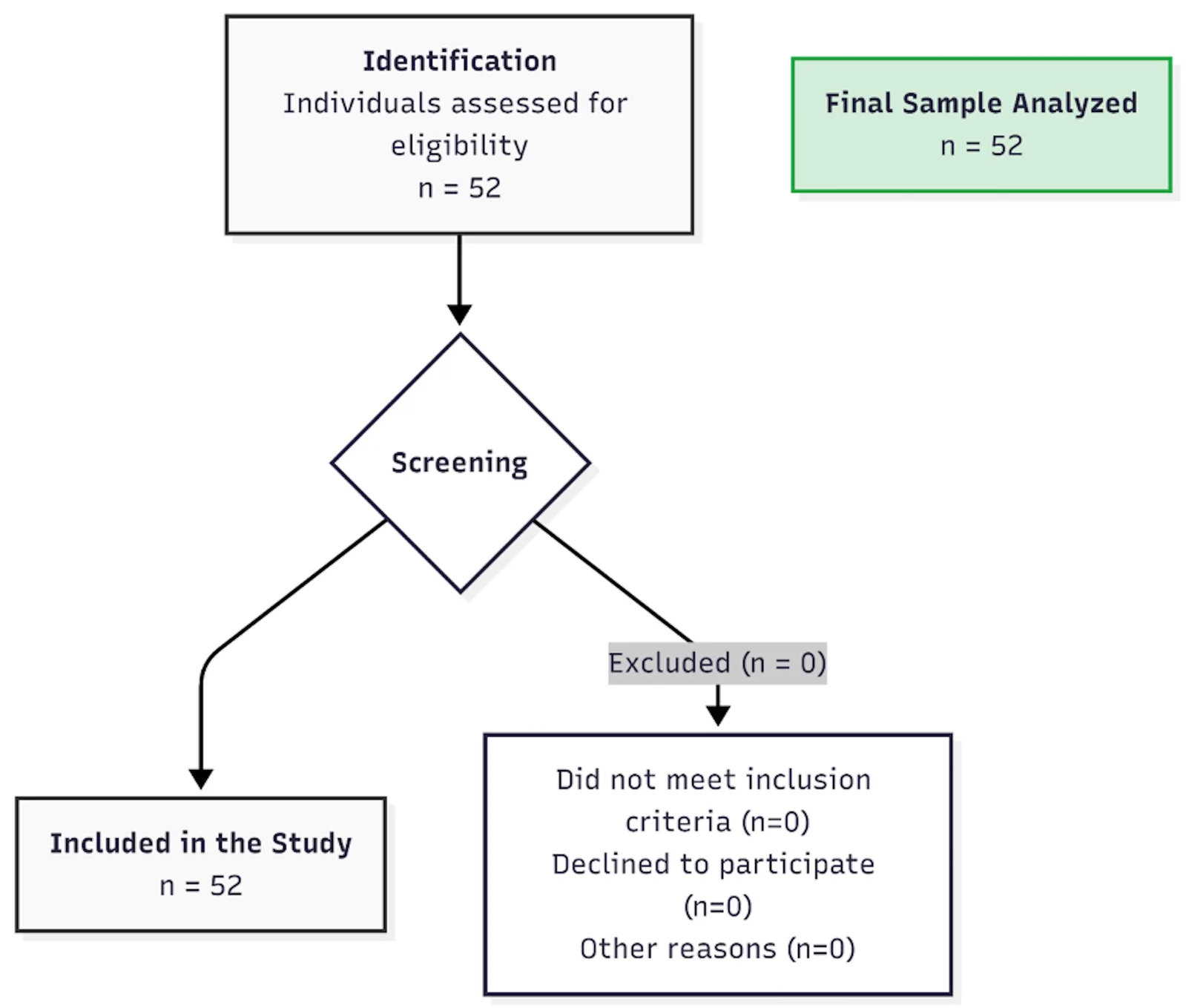
Flowchart of participant selection.

**Table 1:** Sample characteristics.

**Variable**	**Entire sample (n=52)**	**Mild CVI - CEAP ≤ 3 (n=38)**	**Severe CVI - CEAP ≥ 4 (n=14)**	**p-value**
Age (years)	67.2±13.2	67.1±14.5	67.7±10.6	0.393
Female, n (%)	47 (88.7)	33 (86.8)	13 (92.8)	0.481
HRT (repetitions)	41.3±21.1	44.2±22.0	33.9±17.0	0.120
HRT (seconds)	42.4±17.2	43.5±18.5	39.7±13.6	0.493
STS5 (seconds)	13.3 (6.0 – 23.2)	13.4 (11.0 – 17.8)	15.8 (11.7 – 21.6)	0.166
STS10 (seconds)	27.5 (17.0 – 50.6)	27.1 (23.0 – 35.4)	30.8 (25.3 – 39.9)	0.145
STS30 (repetitions)	11.7 (6.0 – 18.0)	11.0 (8.0 – 14.0)	9.5 (7.0 – 11.5)	0.095
STS60 (repetitions)	22.8 (13.0 – 32.0)	21.5 (18.0 – 26.0)	18.5 (14.7 – 22.0)	0.208
Handgrip dynamometry (kg)	28.3 (15.0 – 56.0)	28.0 (26.0 – 34.0)	30.0 (24.5 – 36.5)	0.292
HAP maximum (score)	59.3 (8.0 – 80.0)	56.0 (44.0 – 67.0)	52.5 (39.5 – 59.0)	**0.042**
CIVIQ-14^*^	35.8±13.6	35.21±4.7	37.8±12.8	0.682

Data presented as mean and standard deviation, median and interquartile range, or absolute number and percentage. Abbreviations: CEAP = Clinical (C), Etiological (E), Anatomical (A), and Pathophysiological (P); HRT = Heel-Rise Test; STS = sit-to-stand test; HAP = Human Activity Profile.

* Data collected from 22 patients.

The number of HRT repetitions was correlated with STS5, STS10, STS30, STS60, handgrip dynamometry, and maximum HAP results. However, HRT time did not correlate with handgrip strength as measured by dynamometry. Furthermore, the CIVIQ-14 quality of life questionnaire score correlated with HRT time and repetitions (Table 2).

**Table 2:** Correlations between strength and functionality variables and the number of repetitions and time spent performing the Heel-Rise Test.

	**HRT repetitions**		**HRT time**	
	**r**	**p-value**	**r**	**p-value**
STS5	-0.676	0.001*	-0.386	0.005*
STS10	-0.701	0.001*	-0.399	0.003*
STS30	0.691	0.001*	0.347	0.012*
STS60	0.697	0.001*	0.347	0.013*
Handgrip dynamometry	0.275	0.049*	0.236	0.092
HAP maximum	0.457	0.001^*^	0.427	0.002*
CIVIQ-14	-0.646	0.001*	-0.407	0.060

* Values showed statistical significance.

Comparison of HRT performance between CEAP classes found no differences between patients with severe (CEAP ≥4) and mild disease (CEAP < 4) in number of repetitions (p = 0.120) or time taken (p = 0.493). The performance of patients in the mild disease group varied widely, with some individuals achieving very high values, which may have influenced the lack of significant differences between groups (Figure 3).

**Figure 3:**
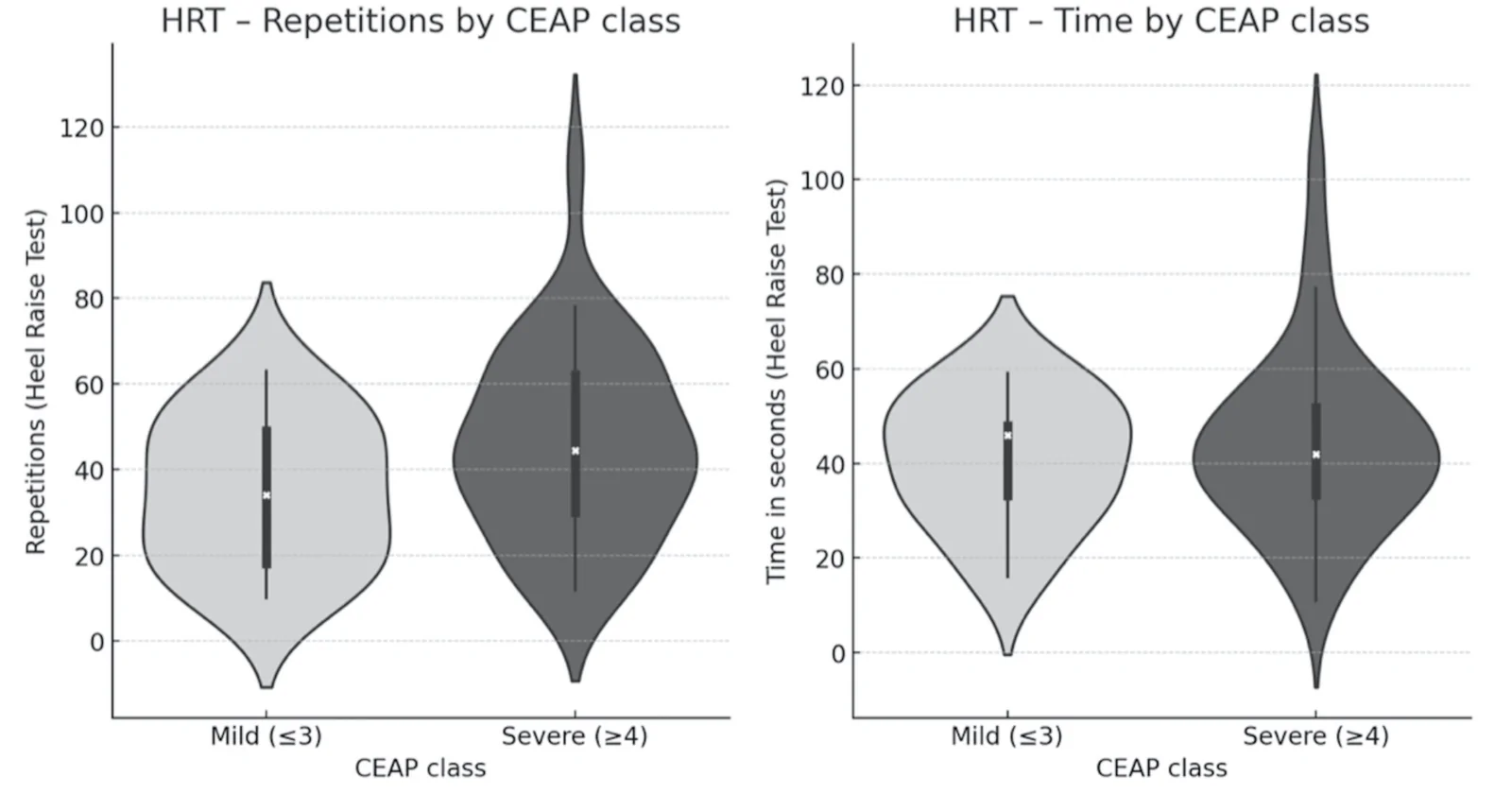
Violin plots of Heel Rise Test (HRT) performance, expressed as repetitions and time, comparing patients with mild (CEAP ≤3) and severe (CEAP ≥4) disease.

## DISCUSSION

The present study verified the applicability of the HRT in patients with CVI, focusing on its correlations with other functional parameters. The main finding of this study was a strong correlation between the number of repetitions and the duration of the HRT, as well as with several functional assessments, including STS tests, handgrip dynamometry, and HAP. From a clinical perspective, these results demonstrate that the HRT is a simple, low-cost, and feasible tool for evaluating calf muscle performance, a key component in the pathophysiology of CVI.

The HRT is a functional assessment commonly administered to patients with venous dysfunction. A previous study^7^ has shown that patients with CVI perform significantly fewer repetitions than healthy individuals (p=0.003). Furthermore, differences in number of repetitions have also been observed when comparing patients with mild and severe CVI (p = 0.040). These findings support the hypothesis that patients with CVI have reduced calf muscle strength and that the HRT is a valuable tool for assessing this impairment.^11^

The relationship between calf muscle function and CVI is likely bidirectional and multifactorial. On one hand, impaired plantar flexor strength and endurance can reduce the efficacy of the calf muscle pump, leading to diminished venous ejection and increased ambulatory venous pressure, ultimately favoring the development or worsening of venous hypertension.^24^ From this perspective, muscle weakness may be considered a potential contributing cause of venous dysfunction. On the other hand, chronic venous hypertension and venous microangiopathy can promote edema, pain, and local inflammatory and structural changes in the muscle–tendon unit, which in turn may reduce muscle performance,^25^ suggesting that muscle impairment can also be a consequence of long-standing CVI. Our findings support an association between poorer HRT performance and more advanced clinical stages of CVI, but longitudinal studies are needed to clarify whether improving calf muscle function can modify the progression of venous disease.

The present study demonstrated a significant correlation between the number of HRT repetitions and STS results (STS5, STS10, STS30, and STS60), which are established measures of lower-limb strength and muscular endurance. This finding demonstrates the construct validity of the HRT, suggesting that it reliably reflects calf muscle performance in a manner consistent with other functional tests. Importantly, the HRT is simpler and requires less equipment and space than the STS, which highlights its practicality as a bedside assessment. Clinically, this means that the HRT may serve as a practical alternative or complementary tool to the STS for evaluating lower-limb function in patients with CVI, particularly in settings in which time or resources are limited.

Furthermore, the number of repetitions also correlated with the HAP score. The moderate correlation observed in our study suggests that greater strength and endurance of the calf muscles, as reflected by HRT repetitions, are associated with higher daily activity levels. This association is physiologically plausible, since the calf muscle pump is a central determinant of venous return during ambulation and reduced muscle performance can limit mobility, decrease activity levels, and ultimately worsen venous hypertension. Supporting this interpretation, Pereira et al.^13^ demonstrated that, taken together, HRT repetitions, level of physical activity, and age explained 47% of the variation in CEAP class, highlighting the clinical relevance of these variables for disease severity. These findings lend support to the idea that HRT repetitions are not only a marker of local muscle function but also reflect broader aspects of functional capacity and disease progression in CVI.

Additionally, HRT repetitions were correlated with quality of life, as assessed by the CIVIQ-14 questionnaire. Although this analysis was conducted in a smaller subsample of 22 patients, the finding nevertheless reinforces the clinical importance of calf muscle performance in CVI. The questionnaire’s domains capture the physical, psychological, and social impact of venous disease, including symptoms such as pain, heaviness, and fatigue, as well as limitations to mobility and daily activities. Therefore, the observed correlation suggests that reduced strength and endurance of the calf muscles, as evidenced by lower HRT repetitions, contribute to the pathophysiology of venous hypertension and culminate in worse patient-reported outcomes. Even though the sample size was limited, this result nevertheless highlights a potential role for the HRT as a functional marker that bridges objective muscular performance and subjective aspects of disease burden. Larger studies are needed to confirm this association and explore whether HRT could be adopted as a surrogate endpoint for quality of life in clinical practice and research.

In contrast to the other functional measures, handgrip dynamometry had a weak correlation with HRT repetitions and no correlation with HRT time. While handgrip strength is often used as a surrogate marker of overall muscular strength,^26^ it does not explicitly reflect the performance of the calf muscles, which play a central role in venous return. This finding suggests that global measures of upper-limb strength may not be adequate proxies for lower-limb muscle function in patients with CVI. Instead, tests such as the HRT, that directly engage the calf muscle pump, appear to provide a more valid and clinically relevant assessment of the functional impairment associated with venous disease.

The lack of statistically significant differences in HRT performance across CEAP clinical classes should be interpreted with caution. First, CEAP is a clinical classification based on observable signs and symptoms and does not directly quantify the functional performance of the calf muscle pump. Therefore, patients classified in different CEAP stages may show overlapping levels of muscle strength and endurance. Second, it is plausible that some individuals in the early clinical stages present with substantial calf pump dysfunction, whereas others in more advanced stages retain preserved or compensatory muscle function, partly due to differences in physical conditioning, obesity, or comorbidities. Third, our subgroup analyses may have been underpowered to detect small to moderate differences, given the relatively modest sample size in some CEAP categories. Altogether, these factors suggest that CEAP class and HRT performance capture related but not identical dimensions of disease burden, one primarily clinical and the other functional, which may explain the lack of clear between-group differences in our sample.

One unexpected finding of this study was the difference in performance between the two HRT outcomes. While the number of repetitions consistently correlated with lower-limb strength, physical activity, and quality of life, test duration had weaker or absent associations, including with handgrip dynamometry. Although not the study’s primary aim, this result suggests that repetitions may represent a more robust and physiologically meaningful outcome than duration, as they directly reflect calf muscle strength and endurance. Conversely, test duration may be influenced by execution pace and patient strategy, which could explain its lower discriminatory power.

Calf muscle pump deficiency is associated with reduced plantar flexor strength and decreased range of motion (ROM) in dorsiflexion and plantar flexion.^9,10^ Plantar flexor muscles undergo several changes in CVI, including a decrease in the number of fast-twitch fibers (type II) and reduced force-generating capacity, as well as morphological and metabolic changes to muscle fibers.^27,28^ These characteristics may, at least in part, explain the greater susceptibility of the calf musculature to fatigue and functional decline observed in patients with CVI. In contrast, handgrip strength primarily reflects the condition of muscles predominantly composed of type I fibers, which are more resistant to fatigue, a fact that may explain the weak correlation found between handgrip dynamometry and HRT outcomes, since handgrip does not specifically reflect functional impairment of the calf musculature, which plays a central role in venous return.

An important conceptual issue is the distinction between “true” venous insufficiency, defined by anatomical or valvular dysfunction, and isolated calf pump impairment or limited plantar flexion, which may produce similar clinical complaints. In our study, all participants had a previous diagnosis of CVI established by an angiologist and venous ultrasound had been performed as part of their routine care, but duplex findings were not systematically recorded for research purposes. Consequently, the HRT in this sample should be interpreted as a measure of calf muscle performance within a population with established venous disease, not as a stand-alone diagnostic test for venous insufficiency. Moreover, although we excluded conditions that could markedly interfere with test performance (such as heel spurs, plantar fasciitis, knee osteoarthritis, and stroke), we cannot completely rule out the contribution to HRT performance made by musculoskeletal factors including reduced ankle range of motion or age-related sarcopenia. This supports the idea that the HRT captures the functional status of the calf pump, which is a key determinant of venous return, but does not differentiate between primary venous abnormalities and isolated mechanical or muscular limitations.

Beyond venous abnormalities, several patient-related factors may also influence calf muscle performance and HRT results. Obesity increases mechanical load on the lower limbs and is frequently associated with reduced ankle range of motion and physical inactivity.^29^ Likewise, low levels of habitual physical activity and unfavorable health behaviors are known to impair muscle strength and endurance.^30^ These factors can contribute to reduced heel-rise performance independently of CVI and may partly explain the variability in HRT outcomes. Thus, while our results support the use of the HRT as a functional measure of calf pump performance in patients with CVI, they also highlight that HRT performance is not specific to venous disease and is modulated by general physical condition.

From the perspective of rehabilitation, the HRT appears to be a promising tool for monitoring functional progress in patients on exercise programs that target the calf muscle pump.^31-33^ However, an important gap remains: most functional tests administered to CVI patients were originally developed and validated in other populations, without accounting for the specific pathophysiological characteristics of venous disease. Therefore, future studies should focus on continued validation of the functional tests, specifically in CVI, as well as on exploring longitudinal outcomes that capture the impact of the disease and treatment response.

Although the present study highlighted the applicability of the HRT, some limitations should be acknowledged. The sample was primarily composed of female patients, which may limit generalization of the results. However, the significantly higher prevalence in females is one of the characteristics of the disease.^34^ In addition, most participants were at less severe stages of CVI, which may have influenced the sensitivity of the HRT to differentiate the most compromised patients. Another relevant limitation to acknowledge is that only 22 of the 52 participants in the present study completed the CIVIQ-14 questionnaire, which limits the scope of the quality-of-life analyses. However, this is a secondary, exploratory outcome, whereas the main conclusions of the study are based on functional measures of calf muscle performance obtained from the entire sample. Another limitation of the study is the lack of systematically recorded Doppler ultrasound data. Although all patients had previously undergone a vascular ultrasound scan conducted by the angiologist who referred them to our service, these findings were not tabulated and therefore could not be included in the analysis. However, since the primary objective of this investigation was a functional assessment of calf muscle performance rather than a hemodynamic characterization of venous disease, we believe that the absence of Doppler ultrasound data does not invalidate the main findings of the present study, although it does eliminate the possibility of correlating functional impairments with specific venous abnormalities.

On the other hand, the study’s main strength lies in the use of a practical, accessible, and easy-to-perform test, with potential for use in various clinical and rehabilitation settings. The simplicity of the HRT makes it a valuable tool, especially in settings in which more sophisticated methods are unavailable. Therefore, the present study contributes to the literature by demonstrating that the HRT can be used as a functional indicator in patients with CVI. Despite its limitations, the HRT constitutes a viable option for supplementing the clinical and functional assessment of these patients, particularly in the early and moderate stages of the disease.

## CONCLUSION

The HRT is a valid and clinically relevant measure of calf muscle performance in patients with CVI. HRT showed consistent correlations with established functional tests of lower-limb strength and endurance, as well as with physical activity levels and quality of life. Although the HRT did not significantly differentiate between mild and severe CEAP classes, it revealed considerable heterogeneity among patients in the early stages of the disease. These findings indicate that the HRT is a simple, low-cost, and feasible tool that can capture functional impairment in CVI, with number of repetitions being the most robust outcome.

## Data Availability

The data supporting this study are available upon request from the corresponding author, HSC. Access may be granted subject to adequate justification and applicable restrictions, including ethical and/or privacy requirements.
